# Synthesis and characterization of growth factor free nanoengineered bioactive scaffolds for bone tissue engineering

**DOI:** 10.1186/s13036-022-00303-x

**Published:** 2022-10-17

**Authors:** Fatemeh Abedi, Sevil Vaghefi Moghaddam, Parisa Ghandforoushan, Marziyeh Aghazadeh, Hafez Ebadi, Soodabeh Davaran

**Affiliations:** 1 Clinical Research Development, Unit of Tabriz Valiasr Hospital, Tabriz University of Medical Sciences, Tabriz, Iran; 2 Drug Applied Research Center, Tabriz University of Medical Sciences, Tabriz, Iran; 3 Department of Medicinal chemistry, Faculty of Pharmacy, Tabriz University of Medical Science, Tabriz, Iran; 4 Stem Cell Research Center and Oral Medicine Department of Dental Faculty, Tabriz University of Medical Sciences, Tabriz, Iran; 5 Department of Materials Engineering, Faculty of Mechanical Engineering, University of Tabriz, Tabriz, Iran

**Keywords:** Nanocomposite scaffold, PCEC, Fe_3_O_4_, HA, Bone tissue engineering, Scaffold, Growth factor free

## Abstract

**Background:**

To address the obstacles that come with orthopedic surgery for biological graft tissues, including immune rejections, bacterial infections, and weak osseointegration, bioactive nanocomposites have been used as an alternative for bone grafting since they can mimic the biological and mechanical properties of the native bone. Among them, PCL-PEG-PCL (PCEC) copolymer has gained much attention for bone tissue engineering as a result of its biocompatibility and ability for osteogenesis.

**Methods:**

Here, we designed a growth factor-free nanoengineered scaffold based on the incorporation of Fe_3_O_4_ and hydroxyapatite (HA) nanoparticles into the PCL-PEG-PCL/Gelatin (PCEC/Gel) nanocomposite. We characterized different formulations of nanocomposite scaffolds in terms of physicochemical properties. Also, the mechanical property and specific surface area of the prepared scaffolds, as well as their feasibility for human dental pulp stem cells (hDPSCs) adhesion were assessed.

**Results:**

The results of in vitro cell culture study revealed that the PCEC/Gel Fe_3_O_4_&HA scaffold could promote osteogenesis in comparison with the bare scaffold, which confirmed the positive effect of the Fe_3_O_4_ and HA nanoparticles in the osteogenic differentiation of hDPSCs.

**Conclusion:**

The incorporation of Fe_3_O_4_ and HA with PCEC/gelatin could enhance osteogenic differentiation of hDPSCs for possible substitution of bone grafting tissue.

**Graphical Abstract:**

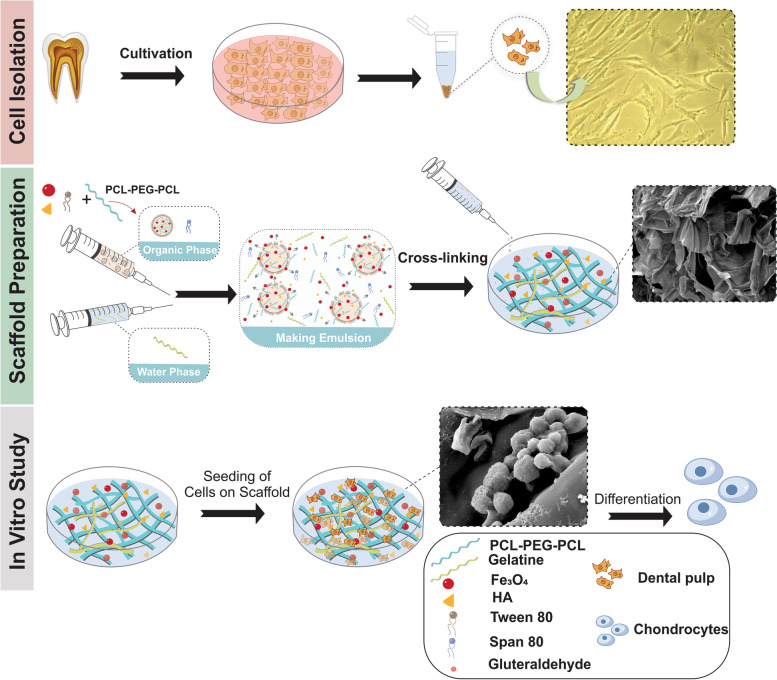

## Introduction

Tissue engineering (TE) is an interdisciplinary science that focuses on utilizing the combination of cells, engineering, and biomaterials along with some appropriate growth factors to repair or replace the injured tissues including skin, bone, and cartilage [1, 2]. Progress in proper cell application, cell culture, and advanced formulation of biomaterials resulted in more effective therapies in tissue engineering and regenerative medicine (TERM). The main goal in this field is the development of biological alternatives to regenerate, preserve, or improve damaged tissue and organ function [3]. Creating tissues with desirable characteristics outside the patient’s body, and using scaffolds and living cells as structural units are other goals of this field. The use of biodegradable polymeric scaffolds to form target tissues is a solution to some medical problems such as tissue loss and organ failure [4]. Generally, scaffolds are degradable polymers with porous architectures, which are mainly used for tissue engineering [5]. They are applicable for in vivo systems to repair or replace damaged tissues in the body as well as mechanical support [6]. They can also be used as carriers for growth factors or antibiotics to accelerate tissue growth or healing or prevent infection [7]. Scaffolds are known as extracellular matrix (ECM) components and are considered a framework for attaching cells relevant to the target tissue [8]. The high vulnerability of bone tissues in various accidents has led to extensive research on tissue engineering, focusing on bone tissues [9]. Among body tissues, bone has a high potential for reproduction and is thus a good sample for tissue engineering [10, 11]. Examining the structures and functions of osteogenic cells is the first step in bone tissue engineering. Bone consists of a solid organic mold or matrix named ECM that is reinforced by the deposition of calcium solutes. Due to the important role of the ECM in stem cell fate, several biologically modified biomaterials have been developed based on the functionalization of the bioengineered scaffold with ECM-derived biomolecules. Among different types of osteoconductive biomaterials hydroxyapatite (HA) and iron oxide magnetic nanoparticles (Fe_3_O_4_) have great importance in the growth and nucleation of calcium phosphate crystals [12, 13]. Their calcification capacity causes the formation of the interfacial layer that makes a strong bond with the host bone at the site of the implant and boosts osseointegration [14]. Also, having the properties such as excellent biocompatibility, non-toxicity, and non-inflammatory behavior enable hydroxyapatite as a scaffold in bone tissue engineering. Furthermore, the use of magnetic nanoparticles is an effective idea in bone regeneration due to their capacity to generate bone tissue via osteo-mimetic architecture. The incorporation of Fe_3_O_4_ nanoparticles with the scaffolds dopped with HA nanoparticles can be an ideal composite for bone tissue engineering owing to their excellent potential to be delivered magnetically to the target site, positive effect on the osteoblast cells, good biocompatibility, and non-toxicity [15]. In addition, the osteoconductive property of the scaffolds associated with the magnetic nanoparticles can improve with or without an external magnetic field [16–18]. The ability of the bone tissue to recognition of mechano-electrical conversion lead to the accelerated rate of osteoblast cells growth and differentiation in the presence of magnetic nanoparticles, resulting in enhanced proliferation and expression level of the genes correlated with bone differentiation [19, 20]. Moreover, they can increase hydrophilicity, and mechanical properties as well as the degradation rate of the scaffolds [20].

Many degradable polymers and polyesters have been used to create a scaffold. α-ester or aliphatic (ε-caprolactone) polyesters and their synthesized copolymers are the most common synthetic degradable polymers that are widely used for medical applications [21]. Poly (ε-caprolactone) (PCL) is relatively non-toxic and has sufficient mechanical strength, environmental compatibility, and thermal stability for scaffolding plans. However, a slow degradation rate due to high hydrophobicity and high crystallization is the main limitation of this polymer [22]. Ring-opening polymerization is the most common method for the synthesis of these copolymers and obtaining products with high molecular weights with controlled microstructures [23]. In this regard, stannous octoate (SnO_2_) is commonly used as a catalyst for the polymerization of cyclic esters due to its non-toxicity and high efficiency.

Hydrogels are three-dimensional biomaterials composed of polymers cross-linked together to form a matrix with tunable properties [24]. In comparison with rigid hard scaffolds which need preshaping, soft scaffolds are injectable and fill any shape defects. Previous groups have reported the fabrication of hydrogels composed of methacrylated glycol chitosan (MeGC) [25, 26]. The hydrogels represent proliferation and ECM deposition of the encapsulated mesenchymal stem cells, but minimal osteogenic ability in the absence of growth factors or bioactive molecules and encapsulated cells due to the lack of porous structures. The authors suggest that porosity is an important factor in forming new tissue since it allows cell migration and proliferation in a three-dimensional environment and facilitates differentiation and vascularization. So, developing a microporous interconnected hydrogel system with the ability of native cells adhesion and bone-forming acceleration could be of great importance. On the other hand, by considering the advantages of the interconnected hydrogels another group reported the highly compatible hydrogels incorporated with different formulations of bioactive glasses (BGs) as scaffolds [27]; but toxic cross-linker and UV light in the polymerization process cause several safety concerns. To address the above-mentioned problems, we engineered composite hydrogels with intrinsic bioactivity and biocompatibility. For this, we designed nanocomposite hydrogel PCL-PEG-PCL/gelatin (PCEC/Gel) incorporated with the HA and Fe_3_O_4_ nanoparticles. We also characterized the physicochemical, mechanical properties, porosity, and swelling behavior of the composite scaffolds as well as the in vitro cytotoxicity and bioactivity. Finally, we evaluated the effect of magnetic nanocomposite on the differentiation of dental pulp stem cells cultured onto the prepared scaffolds. Our results confirm the significant potential of the prepared hydrogels in developing scaffolds with intrinsic biocompatibility and osteoconductivity, which are suitable for bone tissue engineering.

## Methods and materials

Ferric chloride hexahydrate (FeCl_3_,6H_2_O), ferrous chloride tetrahydrate (FeCl_2_,4H_2_O), and ammonium hydroxide (32 wt.%) were purchased from Fluka (Buchs, Switzerland). Gelation (porcine skin, type A), polyvinyl alcohol (PVA), stannous 2-ethyl hexanoate (stannous octoate (Sn (Oct)_2_), toluidine blue, and other chemicals and reagents for the synthesis were purchased from Sigma-Aldrich Co. unless otherwise noted. All the solvents were purchased from Merck Inc. and used without any purification. MTT (3-(4,5-dimethylthiazol-2-yl)-2,5- diphenyltetrazolium bromide) was acquired from Sigma-Aldrich. Dulbecco’s modified Eagle’s medium (DMEM), fetal bovine serum (FBS), and trypsin−EDTA were purchased from Gibco.

### Synthesis of magnetic nanoparticles

The method of preparation was modified from what was reported previously [28]. In a three-necked flask, a mixture solution (100 ml) of FeCl_2_.4H_2_O (4 mmol, 0.76 g) and FeCl_3_.6H_2_O (2.7 mmol, 2 g) was degassed under nitrogen atmosphere at 50 °C for 30 minutes. Subsequently, 20 ml ammonia solution (25% v/v) was immediately added to the homogenous solution to maintain pH at 11 and continue to stir constantly for 40 min. Finally, the temperature of the mixture was raised to 80 °C and the solution was stirred, fastly for 90 min. Following filtration and washing several times with distilled water, the black-colored nanoparticles were obtained and then freeze-dried before further use.

### Synthesis of hydroxyapatite

The solution of (NH_4_)_2_HPO_4_ (20.6 g, 156 mmol) and Ca (NO_3_)_2_.4H_2_O (41.5 g, 176 mmol) was prepared separately. Then, phosphate solution was added to 32 ml simulated body fluid (SBF) solution at 25 °C. The resulting solution was added dropwise (1–2 drops per second) to 50 ml calcium source solution under stirring (500 rpm) at 37 °C and pH 7.4 ± 0.2. The molar ratio of calcium to phosphorus was set at 1.67. After 90 minutes, the resulting solution was kept in an incubator overnight at 37 °C for aging. Subsequently, the product was rinsed with distilled water, then centrifuged, and dried at 80 °C for 24 hours. Finally, the dried product was calcinated in an electrical box furnace at 650 °C for 2 h.

### Synthesis of PCL-PEG_6000_-PCL (PCEC) copolymer

PCEC copolymer was prepared by ring-opening polymerization of ε-caprolactone (ε-CL) using Sn (Oct)_2_ as the catalyst. In summary, ε-CL (7.4 g), PEG (M_n_ = 6000, 0.74 g), and Sn (Oct)_2_ (1 wt%) were added to the reaction vessel under the dry nitrogen atmosphere and continued to stir at 130 °C for 7 h. The obtained polymer was dissolved in dichloromethane (DCM) and reprecipitated in a large amount of cold diethyl ether for purification. The resulting polymer was lyophilized with a freeze dryer (model Christ Alpha 1–4 (USA)) and stored at 4 °C for future use.

### Preparation of PCEC/gel nanocomposite scaffolds containing magnetic nanoparticles and hydroxyapatite

The preparation of the nanocomposite scaffolds consisted of three steps. First, the water-in-oil-in-water (W_1_/O/W_2_) emulsion technique was used for the fabrication of Fe_3_O_4_ and HA-loaded PCEC polymer. Briefly, PCEC (200 mg) was dissolved in dichloromethane (5 cc), then hydroxyapatite (6%w/w) and magnetic nanoparticles (6%w/w) along with Span 80 (1 wt%) were added to the polymer solution under homogenization at 7000 rpm for 3 min to form the W_1_/O emulsion. This emulsion was added to 50 mL PVA (0.5%, w/v) and the mixture was homogenized again at 15,000 rpm. Finally, the resultant nanocapsules were frozen at − 80 °C and lyophilized for 48 h. Second, a suspension of Fe_3_O_4_/HA-loaded polymer, which was prepared by dispersing 200 mg polymer in 5 ml DCM, was added to the aqueous solution of gelatin (5 wt%) in three different groups, which was resulted from dissolving gelatin in distilled water at 40 °C, and then stirred. Third, the nanocomposite scaffolds were prepared by chemical bonding of aqueous gelatin solution with the crosslinker glutaraldehyde as follows. The glutaraldehyde solution (1% v/v) was poured into the mixtures and stirred at 30 °C for 10 minutes. Crossed scaffolds were frozen at − 70 °C and lyophilized by a freeze dryer (Telstar) for 48 h. To remove the remaining aldehyde groups of glutaraldehyde, the scaffolds were immersed in 50 mmol of aqueous glycine solution and incubated at 37 °C for 1.5 hours, rinsed three times with distilled water, and then freeze-dried for the second time.

### Isolation and characterization of hDPSCs

The isolation method and characterization of human dental pulp stem cells (hDPSCs) were described in the previous studies [29, 30]. The scaffolds were immersed in 70% EtOH solution for 20 min, and then they were placed under the UV light for 1 h to sterilize. Afterward, they were washed three times with sterile PBS to eliminate residual EtOH. To remove PBS, they were immersed in 6-well plates in DMEM and incubated at 37 °C for 4 days before cell seeding. After finishing the incubation time, the media of scaffolds was exchanged to chondrogenic media by using FBS (2%, Gibco, Singapore), dexamethasone (100 nM), penicillin (100 μg/mL), *β-Glycerophosphate (0.2%),* and ascorbic acid (50 μg/mL) to facilitate cell differentiation studies. The dental cells were cultured in DMEM containing 10% FBS and 1% Pen-Strep antibiotics and incubated at 37 °C until they reach 80–90% confluency. After that, the culture medium was removed and the cells were washed three times with PBS. Thereafter, they were trypsinized and the cell suspension was added onto the top of scaffolds at a density of 4 × 10^4^ cells per scaffold. To attach cells, the plates were kept in an incubator at 37 °C with 5% CO_2_. To keep cells alive, the cell culture medium was exchanged with fresh media every 3 days during this period. This test was carried out in triplicate for each scaffold.

### Cell proliferation analysis by MTT assay

The survival rate and proliferation of hDPSCs seeded on nanocomposite scaffolds were evaluated using MTT assay. Cells were seeded into 96-well plates at a density of 5 × 10^3^ cells per scaffold and incubated for 3, 7, and 14 days. Following incubation, the culture medium was replaced with a fresh medium containing 200 μL MTT solution (2 mg/mL) and incubated for another 4 h at 37 °C. Then the medium of wells was removed, and 200 μl DMSO was added to each well to dissolve blue formazan crystals. Finally, the absorbance of each well was measured at 570 nm using ELISA plate reader (Multiskan MK3, Thermo Electron Corporation, USA). The viability and proliferation of cells were compared with the control group (hDPSCs seeded directly on tissue culture plates (TCPs)). All scaffolds were analyzed in triplicate and the data were reported as mean ± SD.

### Cell morphology study

To assess the attachment of hDPSCs onto the nanocomposite scaffolds, they were processed and visualized through the FE-SEM method. Briefly, following incubation for 3 and 14 days, the scaffolds were washed twice with PBS for 10 min and fixed in 2.5% glutaraldehyde solution. Subsequently, nanocomposite scaffolds were subjected to dehydration procedure with several concentrations of ethanol (50, 75, 95, and 100) for 15 min and then air-dried at 25 °C. The morphology of scaffolds was monitored by the FE-SEM technique.

### Alizarin red S staining and quantification

The production of calcium deposits was assessed by Alizarin Red S staining protocols. After incubation of scaffolds seeded by hDPSCs in osteogenic media for 21 days, they were washed three times with PBS and fixed in cold ethanol 70% (v/v) for 1 h. The ethanol-fixed scaffolds were rinsed with water and incubated with Alizarin Red S (ARS) solution (40 mM, pH = 4.2) for 20 min at room temperature. To remove excess ARS solution, scaffolds were washed several times with water. Afterward, these scaffolds were destained with 10% (w/v) cetylpyridinium chloride in 10 mM sodium phosphate and left for 15 min at room temperature. Finally, the content of Alizarin Red S was quantified by determining the absorbance at 570 nm.

### Osteogenic-related gene expression

Real-time PCR was done to monitor the transcription level of specific hDPSC genes after cells were exposed to the osteogenic induction medium. Briefly, after 21 days of seeding cells, the total RNA was extracted using Trizol reagent (GENALL) according to the manufacturer’s procedure and quantified by gel-electrophoresis and Nanodrop (Thermo Scientific, Waltham, MA, USA). The total RNA, extracted from each sample was transcripted to cDNA using a RevertAid First Strand cDNA transcription Kit (Fermentase, Life Science, USA). The real-time PCR was performed with a standard 3-step program in lightCycle96® (Roch, Sweden)^.^ The Syber Green Master Mix (Amplion, Denmark) was mixed with cDNA and gene-specific primers of BGLAP, BMP2, RUNX2, SPARC, and GAPDH as house-keeping genes to normalized gene expression levels. The primer sequences (according to previous studies of our group) are listed in Table 1 [29]. The PCR data were assessed by the DDCt procedure.

**Table 1:** List of primers sequences

Gene	Primer	Sequence (5′ → 3′)
BMP2	Forward	ACTCGAAATTCCCCGTGACC
	Reverse	CCACTTCCACCACGAATCCA
BGLAP	Forward	CCACCGAGACACCATGAGAG
	Reverse	GCTTGGACACAAAGGCTGC
RUNX2	Forward	GCGGTGCAAACTTTCTCCAG
	Reverse	TGCTTGCAGCCTTAAATGACTC
SPARC	Forward	GAACCACCACTGCAAACACG
	Reverse	TCATTGCTGCACACCTTCTCA
GAPDH	Forward	ATGGGCAGCCGTTAGGAAAG
	Reverse	ATCACCCGGAGGAGAAATCG

### Determining specifications and features

#### Ft-IR

The functional groups of the synthesized PCL-PEG_6000_-PCL copolymer and the copolymer containing magnetic nanoparticles and hydroxyapatite were examined using the Fourier transform infrared (FT-IR) (Bruker, Germany). The sample was mixed with potassium bromide and pressed to form a tablet. The FT-IR spectrum of the sample was analyzed in the range of 400–4000 cm^− 1^.

#### ^1^H-NMR

Chemical structure of the synthesized PCL-PEG6000-PCL copolymer was recorded on a Bruker AVANCE III 400 MHz (Bruker Daltonics Leipzig, Germany) spectrometer.

#### Thermogravimetric analysis (TGA)

The TGA was performed using (SDTA851, Mettler Toledo) instrument under N_2_ atmosphere from 50 to 700 °C with a heating rate of 10 °C per minute. The initial degradation temperature (T_i_) and the percentage of residual mass were determined through the TGA curve, while the maximum thermal degradation temperature (T_max_) was collected from the maximum peaks of DTG.

#### Brunauer-Emmett-teller (BET)

In this method, the porosity and specific surface area (SSA) of prepared nanocomposite was detriment by quantachrome NOVA (Automated Gas Sorption System, Graz, Austria) instrument. The evaluation of the surface porosity was based on the absorption and desorption amount of N_2_ gas at liquid nitrogen temperature (77 K). Furthermore, the Barrett-Joyner-Halenda (BJH) method was used to calculate the pore volume.

#### Field-emission scanning electron microscopy (FE-SEM)

To assess the morphology, and size of scaffolds, field emission scanning electron microscopy (MIRA3 FEG-SEM, *TESCAN*, voltage of 30 kV) was used. The samples were sputtered by a conducting layer of Au-Pd and analyzed. The pore diameters of scaffolds were identified by Image J software (National Institute of Health, USA).

#### X-ray diffraction (XRD)

The XRD analysis was performed by Bruker D8 Advance AXS Diffractometer, USA using Cu Ka radiation (K = 1.542 A) at speed 1° per min in 2Ɵ range of 7°-80° to study the crystal structure of magnetic nanoparticles of Fe_3_O_4_ and hydroxyapatite (HA).

#### Vibrating-sample magnetometer (VSM)

Magnetic properties of Fe_3_O_4_ nanoparticles and scaffolds containing magnetic nanoparticles were evaluated by a VSM (Meghnatis Daghigh Kavir Co., Iran) at room temperature and by applying 7.5 kW power and 50 V voltage.

#### Mechanical properties

The mechanical properties of PCEC/Gel scaffolds were determined with a universal testing machine (AI-7000-M, Gotech Testing Machine Inc., Taiwan). The standard cylindrical scaffolds (10 mm × 20 mm) were stressed at the strain rate of 5 mm/min. Stress-strain data were calculated by load-displacement measurements in the elastic region of the stress-strain curve.

#### Swelling ratio

To study the water absorption rate of porous scaffolds the swelling ratio was determined as follows: the dry scaffolds in size 1 × 1 × 1 cm^3^ were weighed (W_d_) and soaked in PBS buffer (pH 7.4, 37 °C) for predetermined times. At the end of each time, the scaffolds were taken out and the excess amount of water absorbed on the surface was removed by the filter paper; then they were weighed again (W_w_). The swelling ratio was calculated by the following equation:

$$\mathrm{Swelling}\ \mathrm{ratio}=\frac{W_w-{W}_d}{W_d}$$

### Statistical analysis

Statistical analyses were conducted by applying GraphPad Prism version 8 (GraphPad Software, Inc., La Jolla, CA). All tests were performed in triplicated and represented as mean ± standard deviation (SD) for *n* = 3. Statistical significance between sample groups was assessed using one-way ANOVA analysis and T-test. *P < 0.05 was considered significant, while **P < 0.01, ***P < 0.001, and ****P < 0.0001 are considered highly significant.

## Results and discussion

**Fig. 1:**
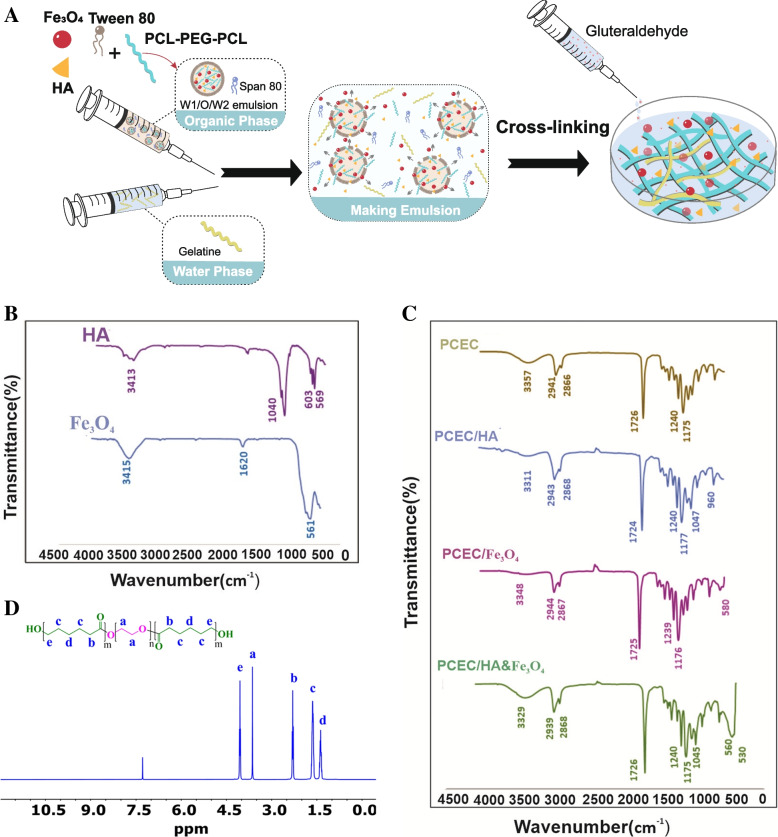
**A** Ischemic illustration for the preparation of PCEC/Gel-Fe_3_O_4_&HA scaffold. FT-IR spectra of **B** HA and Fe_3_O_4_. **C** PCEC, PCEC/Fe_3_O_4_, PCEC/HA, PCEC/Fe_3_O_4_&HA. **D** ^1^HNMR spectroscopy belonging to synthesized PCEC polymer

PCEC/Gel nanocomposite is one of the most commonly used scaffolds for bone tissue engineering as a result of biocompatibility and biodegradability. Herein, we present a proof-of-concept of creating nanocomposite scaffolds based on interconnection between PCEC copolymer and gelatin chains, which doped with hydroxyapatite and superparamagnetic iron oxide nanoparticles. Gelatin is denatured collagen; it has a structure and chemical composition that resembles the extracellular matrix. The studies have shown that HA and Fe_3_O_4_ can enhance the mineralization of the scaffold and have a vital role in the proliferation and differentiation of osteoblasts [31]. Although HA and Fe_3_O_4_ have been used as doping elements in scaffolds for Bone TE, the simultaneous incorporation of them in the PCEC/Gel system has not been reported yet. Here, we try to characterize the physicochemical properties of PCEC copolymer and PCEC/Gel scaffold as well as the feasibility of incorporating HA and Fe_3_O_4_ into the PCEC/Gel to yield the nanocomposite scaffolds for Bone TE. The construction route for the preparation of PCEC/Gel-HA& Fe_3_O_4_ is illustrated in Fig. 1A.

### FT-IR analysis

To evaluate the encapsulation of the HA and Fe_3_O_4_ into the PCEC copolymer FT-IR spectra of the PCEC, PCEC/Fe_3_O_4_, PCEC/HA, PCEC/Fe_3_O_4_&HA was conducted and compared with the spectra of HA and Fe_3_O_4_. As depicted in Fig. 1C the characteristic band at 1740 cm^− 1^ corresponded to C=O stretching of the ester groups. The results revealed that all spectra exhibited the same absorption band. The peaks at 1105 cm^− 1^ and 1169 cm^− 1^ were corresponding to the vibration bands of C-O-C. The stretching vibration of the OH group appeared at 3446 cm^− 1^. The peaks at 2800–2900 cm^− 1^ could be attributed to the aliphatic C-H stretching bonds. In Fig. 1B, in the spectrum of Fe_3_O_4,_ the absorption band at 561 cm^− 1^ belonged to the Fe-O group, which was observed in the spectrum of the PCEC/Fe_3_O_4_ and PCEC/Fe_3_O_4_&HA, indicating the successful incorporation of magnetic nanoparticles with PCEC copolymer. Also, the peaks in 1620 cm^− 1^ attributed to the bending mode of OH groups of adsorbed water [32, 33]. In the spectrum of HA, the peaks located at 569 cm^− 1^ and 603 cm^− 1^ corresponded to the asymmetric and symmetric bending modes of the PO_4_^3−^ group. The appearance of phosphate stretching vibration in the spectrum of the PCEC/HA and PCEC/Fe_3_O_4_&HA also confirmed the successful incorporation of HA with PCEC copolymer. The peak at 1040 cm^− 1^ was attributed to the C-O stretching of the carbonate group which was substituted by the phosphate group [34]. The band at 3413 cm^− 1^ was corresponding to the stretching vibration of O-H groups in the apatite lattice [35].

### ^1^HNMR spectroscopy

The ^1^HNMR spectrum of the PCEC copolymer is depicted in Fig. 1D. The presence of a singlet peak at 3.63 ppm (H_a_) was assigned to the methylene protons of the PEG block in the copolymer. Additional signals at 4 ppm (He), 2.35 ppm (Hb), 1.65 ppm (Hc), and 1.48 ppm (Hd) came from the PCL block in the copolymer chain [36].

### Thermal gravimetric analysis

**Fig. 2:**
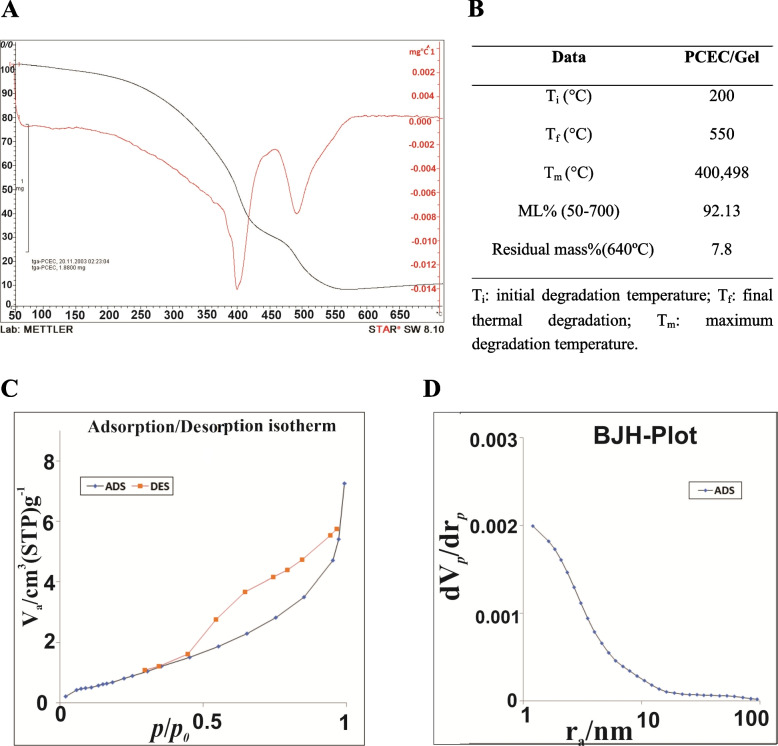
**A** TGA and DTG curves of nanocomposite scaffolds. **B** Thermal parameters derived from TGA and DTG data of nanocomposite scaffolds. Porosity and specific surface area of materials in nanocomposite scaffolds (PCEC/Gel), **C** Adsorption-desorption isotherm, **D** The BJH curve

The thermal stability and degradation behavior of PCEC/Gel nanocomposites were examined by TGA and DTG with a heating rate of 10 °C per minute in the flow of N_2_ gas from 50 °C to 700 °C. As depicted in Fig. 2A, the degradation process of PCEC/Gel nanocomposites have two maximums around 400 °C and 500 °C. The lower degradation temperature, which referred to the greatest reduction in mass, was induced by disintegrating intermolecular interactions as well as the breakdown of the copolymer backbone [37], while the second maximum mostly referred to gelatin decomposition. As shown in the DTG curve, the initial degradation temperature (Ti) of the PCEC/Gel nanocomposites was around 200 °C and the main degradation prosses took place in the range of 200–450 °C and 450–550 °C, corresponding to 66.35 and 20.7% weight loss, respectively [38–40].

### Porosity measurement and BET surface area

The uptake property and adsorbent capacity of the prepared scaffold was predicted from the porosity and specific surface area. In this regard, the Brunauer-Emmett-Teller (BET) theory was used to determine the specific surface areas of PCEC/Gel and the corresponding N_2_ adsorption-desorption curve is shown in Fig. 2C. The obtained isotherm of PCEC/Gel corresponded to type III according to the IUPAC classification. The BET surface area of PCEC/Gel was 3.274 m^2^/g. The pore size distribution was determined by Barrett–Joyner–Hanlenda (BJH) theory and represented the nanoporous structure of PCEC/Gel with the average pore radius of 1.21 nm and a pore volume of 0.011 cm^3^/g. The corresponding pore size distribution is depicted in Fig. 2D, which represented the presence of mesopores and micropores.

### X-ray diffraction (XRD) analysis

**Fig. 3:**
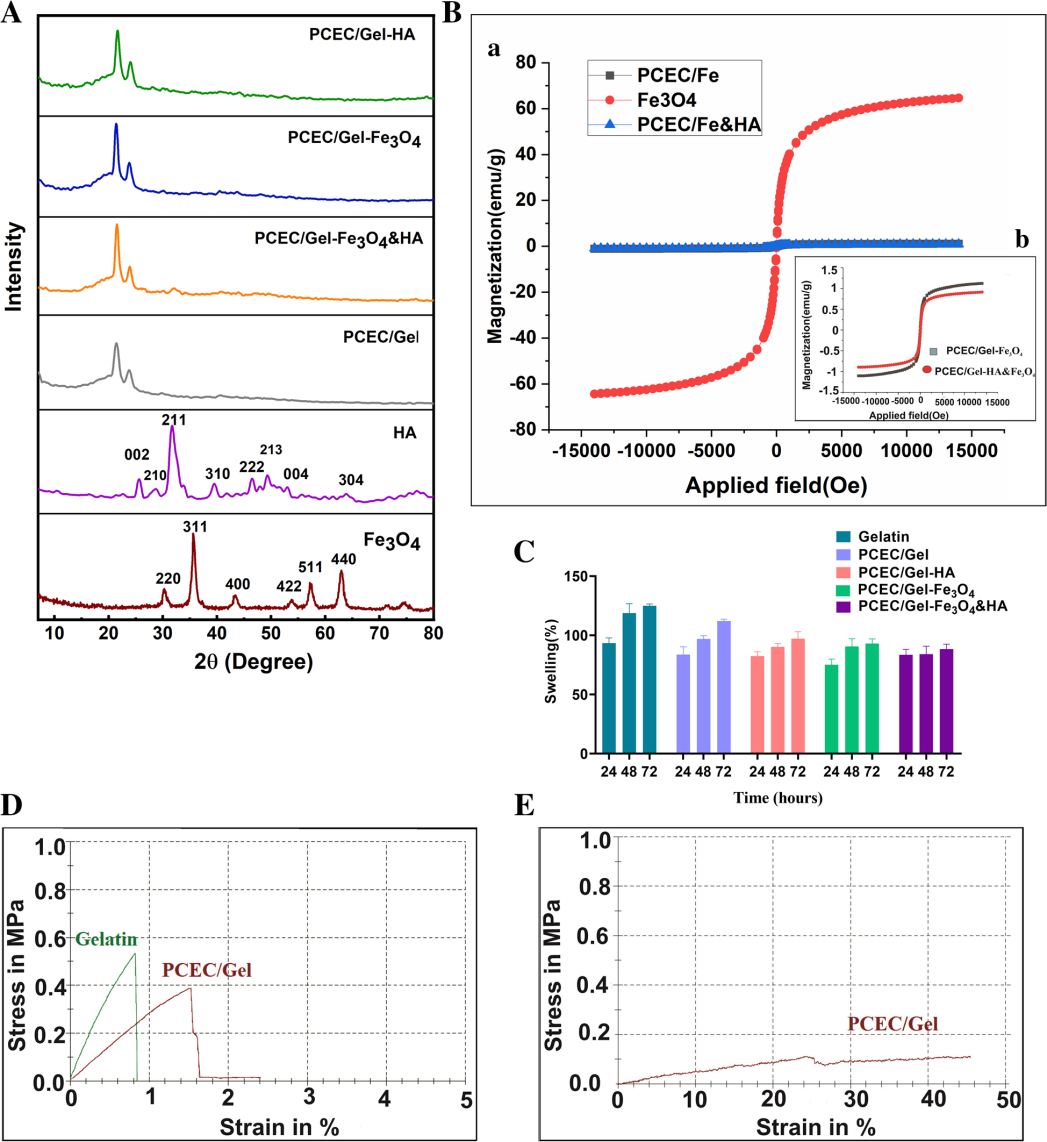
**A** X-ray diffractograms belonging to magnetic nanoparticles, hydroxyapatite, and different groups of nanocomposite scaffolds. **B** Magnetic properties analysis for Fe_3_O_4_ nanoparticles and scaffolds containing magnetic nanoparticles (**a**, **b**). **C** Swelling behavior in gelatin and nanocomposite scaffolds. **D** Stress-strain curves in tensile tests of gelatin and scaffolds in dry condition. **E** Stress-strain curves in tensile tests of scaffolds in wet condition

The X-ray diffraction (XRD) pattern of Fe_3_O_4_ NPs and HA, along with PCEC/Gel nanocomposite scaffolds and scaffolds containing 6% Fe_3_O_4_, and 6% HA, separately and in combination with together were shown in Fig. 3A. The characteristic diffraction peaks, which corresponded to the HA were observed at 2θ angle values of 25.7°, 31.65°, 32.89°, 39.62°, and 49.3° [34, 41]; and the peaks at 2θ of about 30.15°, 35.6°, 43.27°, 57.3°, and 63° were attributed to the reflection plane of Fe_3_O_4_ NPs [42]. The powder x-ray diffraction of the nanocomposite scaffold revealed that the structure is mostly amorphous. Compared to crystalline materials, amorphous materials are more prone to hydrolytic degradation. The high sensitivity of amorphous polymer to hydrolytic degradation is due to the easy transfer of water molecules to the inner region of the polymer that can prove the biodegradability of the copolymer. The peak at 2θ angle around 20° was attributed to the gelatin reflection plane [43], while the peak located at 2θ angle around 23.6° corresponded to the PCL block of the PCEC copolymer, which means that the crystallinity of the PEG block was restricted by the outer PCL block [44]. In the diffractogram of PCEC/Gel-Fe_3_O_4_, the peak at 2θ angle around 25° corresponded to the HA, which confirms the presence of HA in the nanocomposite scaffolds; however, the characteristic peaks of metal oxide nanoparticles were not observed in the diffractogram due to the utilizing negligible percentage for doping, which could be under the detection limit of the apparatus.

### Vibrating-sample magnetometer (VSM)

To measure the magnetic properties of the synthesized samples, the VSM was performed at 25 °C and the corresponding hysteresis curve was prepared by changing the magnetic field of H from − 15,000 to + 15,000 Oe. As shown in Fig. 3B, the saturation magnetization (M_s_) value of Fe_3_O_4_ NPs was 61.54 emu.g^− 1^ at room temperature (300 K), which significantly reduced for the PCEC/Gel-Fe_3_O_4_ and PCEC/Gel-Fe_3_O_4_&HA. Reduction in saturation magnetization of scaffold nanocomposites was due to the use of a negligible amount (6 wt%) of magnetic nanoparticles, but the samples also represented paramagnetism. Hysteresis magnetization and negligible magnetic coercivity in the results indicated that the nanoparticles were superparamagnetic in both samples, and it was proven by converting the hysteresis loop into an S-shaped curve [45, 46].

### Analysis of mechanical properties of nanocomposite scaffolds

As one of the most important biomaterials, gelatin has gained more attention in bone tissue engineering due to the induction of osteogenesis, angiogenesis, and wound healing [24]. However, compared to other polymers like metallic compounds, it has lower loading capacity and elastic moduli. To address the problem, the incorporation of the gelatine with a biocompatible polymer like PCEC could enhance the bioactivity and mechanical property of the resulting scaffold [24]. Determining the mechanical properties of scaffolds is a basic issue in tissue engineering as these properties can affect cell behavior during culturing, adhesion, proliferation, and signaling. Increasing the porosity of the scaffold reduces the mechanical properties [47]. Our results showed that Young’s Moduli increased from 29.44 MPa (pristine gelatine) to 78.79 MPa (PCEC/Gel nanocomposite scaffolds) and the tensile strength increased from 0.39 to 0.53, respectively. Improving the mechanical properties of the nanocomposite scaffold could be explained as a result of decreasing the porosity and increasing the wall thickness of the scaffold pores [48]. The mechanical strength values ​​obtained from hydrated nanocomposite scaffolds were also compared with dry nanocomposite scaffolds. In dry conditions, the values of Young’s modulus and tensile strength increased, but Young’s modulus and tensile stress decreased significantly due to the water absorption. As shown in Fig. 3E, Young’s modulus and tensile strength decreased to 1.29 and 0.11 MPa respectively, but the tensile at the fracture point increased by 45.22%. Hydration of scaffolds led to the plasticity effect, thereby reducing the mechanical properties [49]. Hence, the preparation of PCEC/Gel nanocomposite scaffolds can be considered as an effective scaffold with suitable mechanical and biological properties due to the intrinsic bioactivity of the nanocomposite scaffold, which, in turn could drive diferentation and mineralization of the hDPSCs in vitro.

### Swelling behavior in nanocomposite scaffolds

Despite the advantages that come from the hydrophilic nature of the scaffolding hydrogels, the excess water uptake has been shown to interrupt cell migration and vascularization [50]. Therefore, the swelling ratio is usually used to assess the hydrophilicity, porosity, and pore size of the scaffold. In this regard, we evaluate the swellability of the gelatin, PCEC/Gel, PCEC/Gel-Fe_3_O_4_, PCEC/Gel-HA&Fe_3_O_4_, and PCEC/Gel-HA scaffolds in vitro. The results that were depicted in Fig. 3C, indicated that the gelatin scaffold had a higher water-binding capacity than other scaffolds. Generally, the results suggested that the collaboration of gelatin with PCEC copolymer increased the cross-linking density between polymer chains due to the use of glutaraldehyde cross-linker, which, in turn, decrease the in vitro swelling capacity of the nanocomposite scaffolds. The incorporation of the Fe_3_O_4_ NPs and HA into the PCEC/Gel scaffold decreased the swelling capacity of scaffolds due to the hydrophobic nature of Fe_3_O_4_ NPs and hydroxyapatite [51, 52]. It should be noted that the swelling rate initially increased for all samples. However, it decreased with the continued absorption of PBS molecules and ions. After the first rapid penetration of the solution into the porous structures of nanocomposite scaffolds, the osmotic pressure difference between the samples and the surrounding solution decreased, and the scaffolds began to absorb the solution at a slower rate until they reached equilibrium. Due to the general hydrophilic nature of nanocomposite scaffolds in tissue engineering applications, it is expected that the scaffold hydrophilicity will increase cell transplantation and proliferation at the bone-implant site.

### Evaluation of structures and morphology of magnetic nanoparticles and hydroxyapatite nanoparticles.

**Fig. 4:**
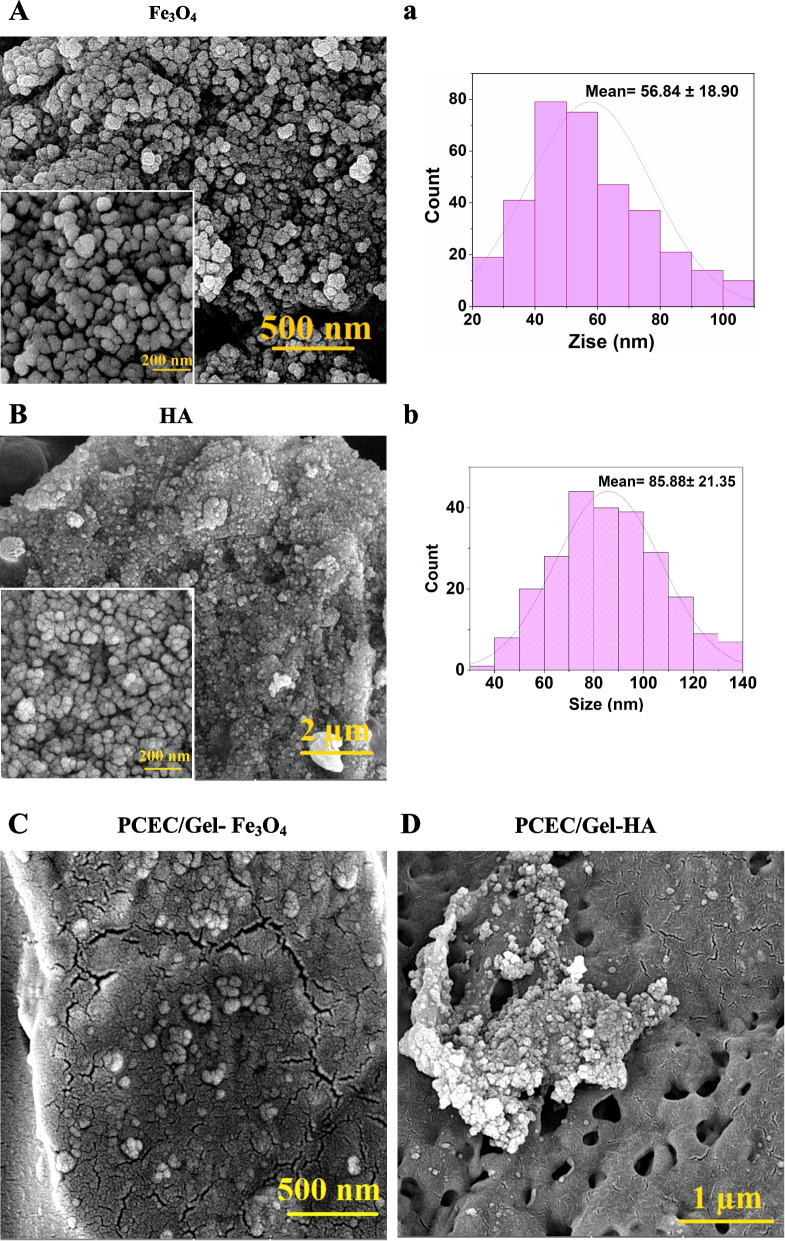
**A**, **a**) Structures and morphology of magnetic nanoparticles and diameter distribution of magnetic nanoparticles. **B**, **b**) Structures and morphology of HA nanoparticles and diameter distribution of hydroxyapatite nanoparticles. **C**, **D**) Structures and morphology of scaffolds containing HA and Fe_3_O_4_

In this section, the morphology of the synthesized Fe_3_O_4_ and HA nanoparticles, dopped into the polymer scaffold as a reinforcing mineral phase, was evaluated. According to Fig. 4A, the average size of magnetic particles was about 56.84, which was calculated using image j software (Fig. 4a). The nanoparticles, which were prepared by the chemical coprecipitation method, were spherical. In Fig. 4B, the SEM micrograph related to the synthesized HA nanoparticles, and represented the average size distribution of about 85.88 nm according to the image j software (Fig. 4b). Figure 4C and D showed the images of magnetic nanoparticles and hydroxyapatite on nanocomposite scaffolds. These nanoparticles were well placed in the scaffolding and spread on the surface of the composites.

### Research on cell compatibility of nanocomposite scaffolds

MTT assay was used to evaluate the biocompatibility of the nanocomposite scaffolds. For this, several groups of scaffolds including gelatin, PCEC/Gel, PCEC/Gel-Fe_3_O_4_, PCEC/Gel-HA, PCEC/Gel-HA&Fe_3_O_4_ were prepared and seeded with the hDPSC cells and cultured for 3, 7, and 14 days. The hDPSCs without scaffold were used as the control group. Cell viability and proliferation of the hDPSCs were determined via the absorption amount of the produced formazan due to the mitochondrial activity of the living cells. Our results represented that nanocomposite scaffolds could support the proliferation of the hDPSC cells as can be seen in Fig. 6A. Also, the results showed that viability and proliferation of the cells increased during 14 days of culture for all groups. By comparing the result of different groups we can see the positive effect of HA and Fe_3_O_4_ NPs incorporated with nanocomposite scaffolds, especially at day 14. The results indicated that hydrophilicity of the mineral nanoparticles facilitates adhesion and then proliferation of the cells over time. The highest viability of the cells incorporated with PCEC/Gel-HA, PCEC/Gel-Fe_3_O_4_, PCEC/Gel-HA&Fe_3_O_4_ scaffolds at day 14 confirmed the effective cell attachment and proliferation during this time. Taken together, the PCEC/Gel-HA&Fe_3_O_4_ scaffolds were non-toxic and presented excellent supports for cell proliferation; this means they can be an ideal candidate for tissue engineering.

### The morphology study of cells cultured on nanocomposite scaffolds

**Fig. 5:**
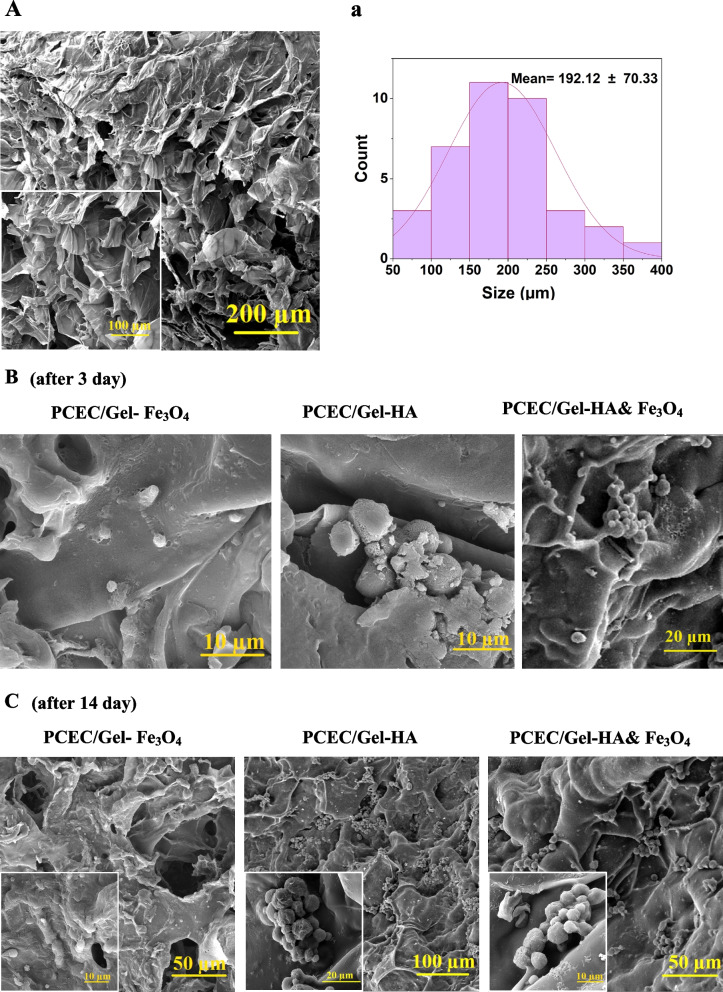
FE-SEM images relating to nanocomposite scaffolds. **A**, **a**) nanocomposite scaffolds before cell implantation, and diagram of the pore size distribution of nanocomposite scaffolds. **B**) hDPSCs cultivated on nanocomposite scaffolds after three days. **C**) hDPSCs cultivated on nanocomposite scaffolds after 14 days

One of the important issues expected from the scaffolding biomaterials is mimicking the porosity and structure of the native bone, which could, in turn, develop cell adhesion along with in vivo tissue ingrowth and vascularization [53]. First, nanocomposite scaffolds were formed and freeze-dried. Then. hDPSCs with the size of about 7–11 μm were cultured on nanocomposite scaffolds for 14 days, and the morphology of attached cells on the scaffolds was observed using SEM images. Figure 5 represented the SEM micrograph of PCEC/Gel-HA, PCEC/Gel-Fe_3_O_4_, PCEC/Gel-HA&Fe_3_O_4_ and confirmed the formation of microstructures and interconnected pores for all samples before and after cell culture. As shown in Fig. 5A, PCEC/Gel had porous structures before cell implantation. We used the freeze dryer method in the formation of porous nanocomposite scaffolds [41]. Porosity is an important factor in cell growth because it provides a proper interaction of the cell with the scaffold. Large pores in the scaffold can support the transformation of nutrients and elimination of metabolic wastes, and thus they are essential for effective cell growth, but they can reduce cell adhesion. Small pores can improve cell adhesion, despite reducing the transfer of nutrients and gas [42, 54]. Hence, the size of a scaffold pore is an important factor. It must be large enough for nutrients to be released and for cells to migrate, and it must also be small enough for cells to have the right area to attach [55]. Scaffolds in a size of 325–100 μm are suitable for tissue engineering [56, 57]. The micro-pores in the prepared porous scaffolds have a size in the range of 192.14 ± 70.33 μm, indicating the best adhesion. Figure 5B, C, and D show the attachment of cells and their random distribution on the surfaces of scaffolds. The cells could disperse and distribute properly and sufficiently on the surface of the scaffolds and fill the pores. Furthermore, there was no delay or inhibition of dental cell proliferation by scaffolds after 14 days due to the non-toxicity of scaffold components and their favorable conditions for cell attachment and growth [58]. It also seems that nanoparticles provide better space for cells and better interaction of scaffolds with cells due to having a larger surface area to volume ratio [56, 58].

### Alizarin red S test

**Fig. 6:**
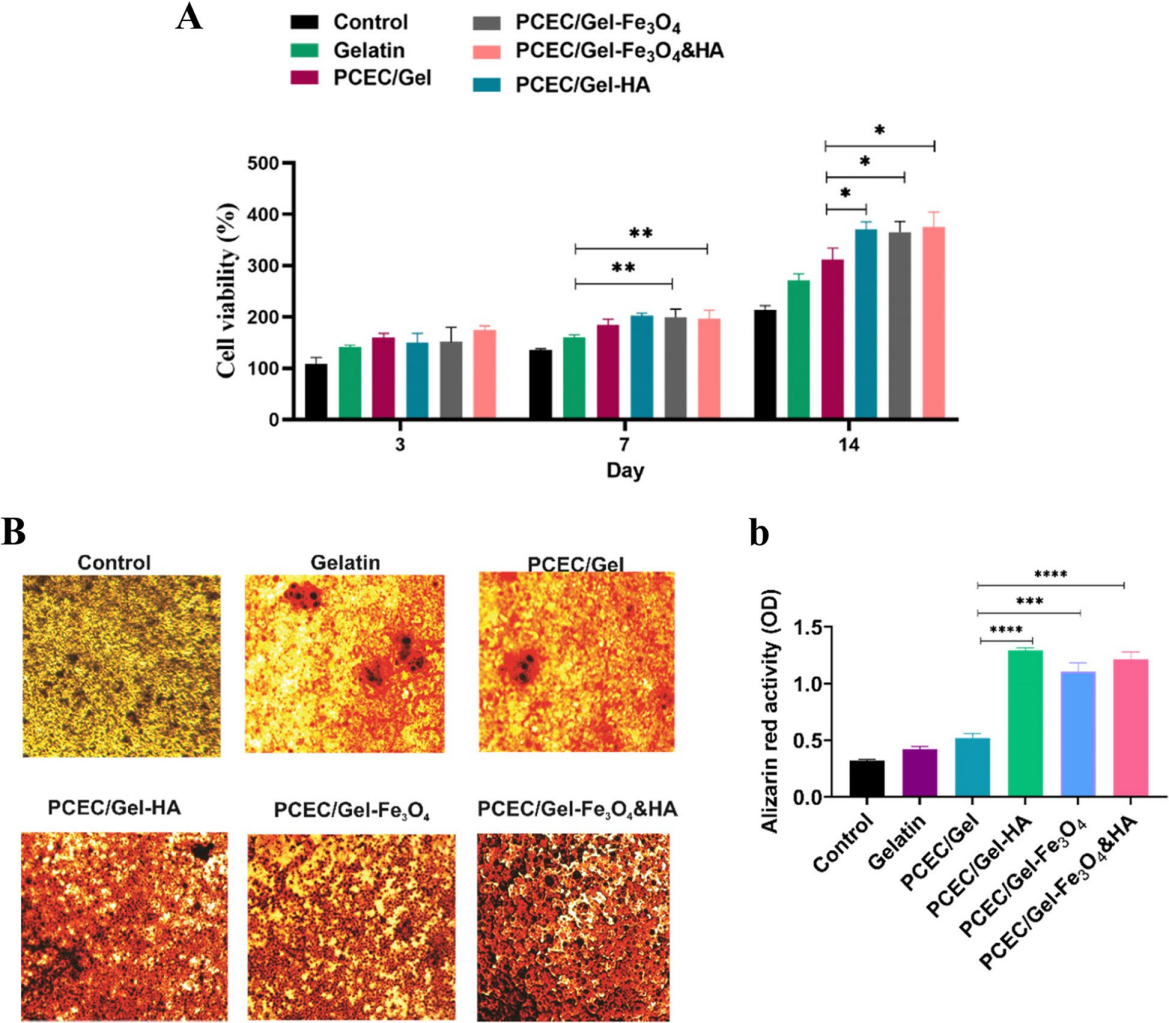
**A**) MTT assay of hDPSCs cultivated on Gelatin, PCEC/Gel, PCEC/Gel-Fe_3_O_4_, PCEC/Gel-HA, PCEC/Gel-HA&F_3_O_4_ scaffolds. **B**, **b**) Alizarin Red staining was performed on day 14 to observe calcium and mineral deposits in nanocomposite scaffolds. The amount of Alizarin Red staining in different compounds was drawn with Prism software. The groups were compared by the one-way ANOVA, and they were then analyzed using the Tukey’s HSD, *p < 0.05, **p < 0.01, ***p < 0.001, and ****p < 0.0001

Histological assessment for several groups of nanocomposite scaffolds using Alizarin Red S staining was shown in Fig. 6B. Differentiation of hDPSCs to the osteoblast can be evaluated by the calcium deposition. In the presence of calcium, Alizarin Red S binds to it and forms a pigment that is in orange to red [59]. The hDPSCs were cultured for 14 days for quantification with Alizarin Red S staining. The Quantitative measurements indicated that cells treated with PCEC/Gel-HA, PCEC/Gel-Fe_3_O_4_, PCEC/Gel-HA&Fe_3_O_4_ exhibited a higher level of calcium deposition in comparison with pristine gelatine, PCEC/Gel, and control group. Also based on the above-mentioned morphological study of the scaffolds, we can explain that the weaker structural integrity and larger pore size of the pristine gelatine caused minimal calcium deposition, while smaller pore size of the PCEC/Gel-HA induced significant calcium deposition as demonstrated in Fig. 6B. In addition, the observation of intensified staining in these groups confirmed the positive effect of HA and magnetic nanoparticles on osteogenic differentiation of human dental pulp stem cells. Mineralization refers to the extracellular deposition of calcium and phosphate ions, which ultimately leads to calcification and is important for bone regeneration. Therefore, hydroxyapatite and magnetic nanoparticles in nanocomposite scaffolds may play an important role in stimulating mineralization.

### qRT-PCR analysis

**Fig. 7:**
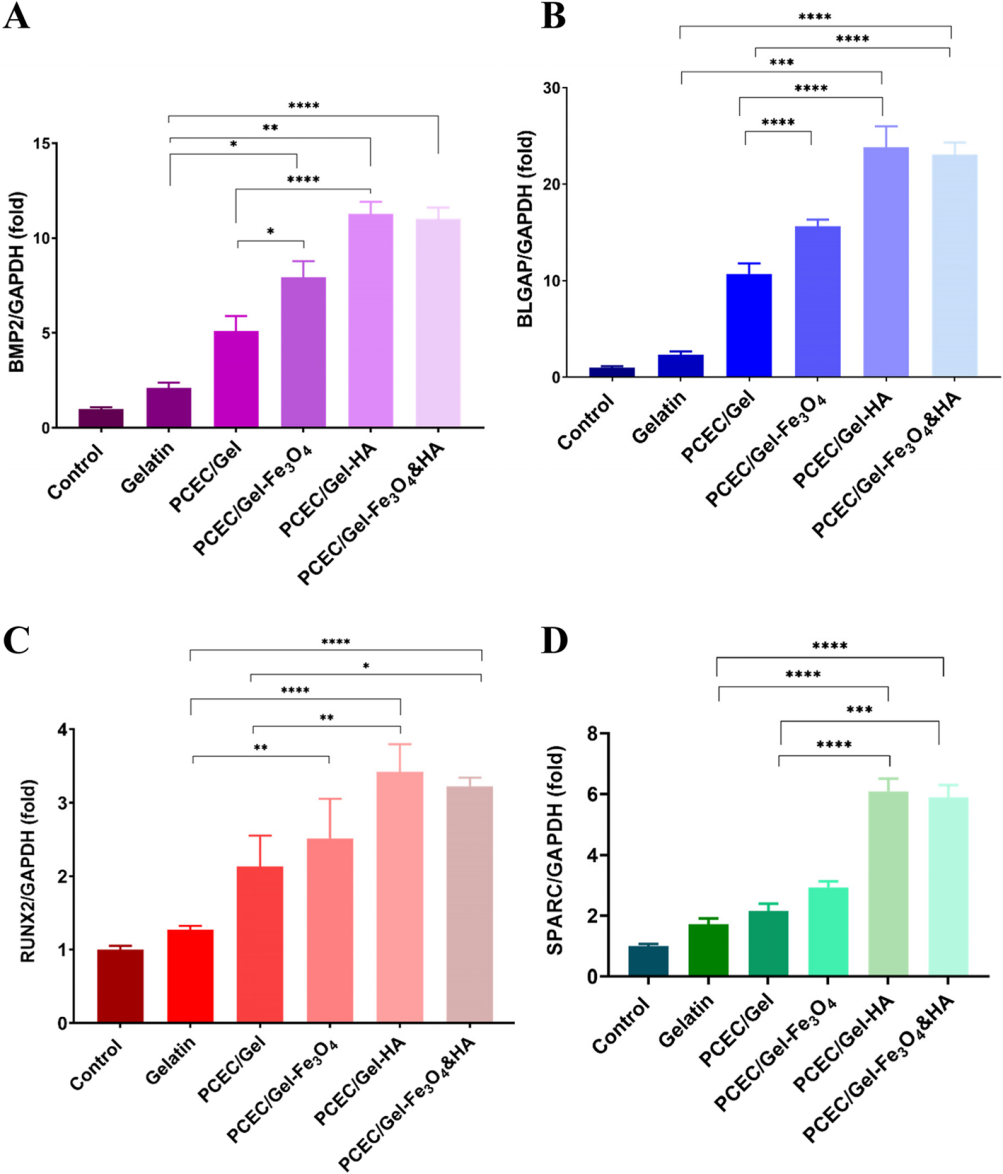
The expression of osteogenic-related genes. Relative expression of **A** BMP2, **B** BGLAP, **C** RUNX2 and **D** SPARC by hDPSCs seeded on gelatin, PCEC/Gel, PCEC/Gel-Fe_3_O_4_, PCEC/Gel-HA, PCEC/Gel-Fe_3_O_4_&HA scaffolds after 21 days by real-time PCR analysis. The values were normalized to GAPDH. (*p < 0.05, **p < 0.01, and ***p < 0.001)

To evaluate the effect of HA and Fe_3_O_4_ on the osteogenesis differentiation level of hDPSCs cultured from the scaffolds, we performed qRT-PCR after 21 days under the osteogenic conditions. As representative osteogenic markers, the gene expression of RUNX2 (the main transcription factor for bone formation), BGLAP (bone mineralization factor), BMP2 (skeletal development and extracellular matrix maturation factor), and SPARC (osteonectin marker) was chosen, which are capable of bone formation and mineralization at the same time. As depicted in Fig. 7 there was no significant difference between the control and gelatin groups, however, the scaffolds containing HA and Fe_3_O_4_ exhibited the highest expression compared to the other type of scaffold and genes, suggesting the optimized concentration of HA and Fe_3_O_4_ incorporated into the nanocomposite scaffolds. In other words, the doping process enhanced the bioactivity for osteogenesis and bone regeneration despite being used in negligible amounts. Considering the aforementioned results, we can speculate that the mineral nanoparticles inside the scaffolds could affect the gene expression of cultured cells compared to the bare PCEC/Gel and gelatin.

## Conclusion

In summary, we have demonstrated the fabrication of interconnected microporous scaffolds using PCL-PEG-PCL copolymer and gelatin chains, which doped with hydroxyapatite and superparamagnetic iron oxide nanoparticles. We observed that the scaffolds could promote cell proliferation as well as calcium deposition in the absence of any growth factors. In addition, PCEC/Gel-Fe_3_O_4_&HA scaffold could promote osteogenesis in comparison with the bare scaffold, which confirmed the positive effect of the Fe_3_O_4_ and HA nanoparticles in the osteogenic differentiation of hDPSCs. This bioactive and biocompatible scaffold can be easily fabricated and might have a niche in tissue regeneration.

## Data Availability

All data generated or analysed during this study are included in this published article.

## Abbreviations

TE: Tissue engineering
PCL-PEG-PCL/Gelatin: PCEC/Gel
HA: Hydroxyapatite
TERM: Tissue engineering and regenerative medicine
ECM: extracellular matrix
hDPSCs: human dental pulp stem cells
PCL: Poly (ε-caprolactone)
MeGC: Methacrylated glycol chitosan
MTT: (3-(4,5-dimethylthiazol-2-yl)-2,5 diphenyltetrazolium bromide
PVA: Polyvinyl alcohol
FBS: Fetal bovine serum
DMEM: Dulbecco’s modified Eagle’s medium
BGs: Bioactive glasses
ARS: Alizarin Red S
DCM: Dichloromethane
hDPSCs: Human dental pulp stem cells
TCPs: Tissue culture plates
FT-IR: Fourier transform infrared
TGA: Thermogravimetric Analysis
BET: Brunauer-Emmett-Teller
XRD: X-ray diffraction
VSM: Vibrating-sample magnetometer
BJH: Arrett–Joyner–Hanlenda
SBF: Simulated body fluid
SSA: Specific surface area
